# Endoscopic ultrasound‐guided treatment for malignant afferent loop obstruction after Roux‐en‐Y reconstruction

**DOI:** 10.1002/deo2.3

**Published:** 2021-02-10

**Authors:** Koichiro Mandai, Koji Uno, Kenjiro Yasuda

**Affiliations:** ^1^ Department of Gastroenterology Kyoto Second Red Cross Hospital Kyoto Japan

**Keywords:** afferent loop obstruction, afferent loop syndrome, endoscopic ultrasound, gastrojejunostomy, stent

## Abstract

The usefulness of endoscopic ultrasound (EUS)‐guided gastrojejunostomy (EUS‐GJ) using a lumen‐apposing metal stent (LAMS) has been reported. However, LAMS is not available in many countries and is more expensive than a conventional fully covered self‐expandable metal stent (FCSEMS). We treated cases of malignant afferent loop obstruction after Roux‐en‐Y reconstruction: three patients underwent EUS‐guided hepaticoenterostomy (EUS‐HES) and one patient underwent EUS‐GJ with a conventional biliary FCSEMS, instead of EUS‐GJ with a LAMS. In two of the cases, EUS‐GJ or EUS‐guided jejunojejunostomy was not indicated because the afferent loop was far from the stomach or jejunum, and EUS‐HES was performed. In one case, in which both EUS‐HES and EUS‐GJ were feasible, EUS‐HES was performed because of unavailability of LAMS for EUS‐GJ in Japan. In another case, EUS‐HES was not indicated because of massive ascites around the liver, and thus, EUS‐GJ using a 10 mm FCSEMS combined with a 7 Fr large‐loop double‐pigtail plastic stent was performed. In all four cases, the patients’ symptoms improved without any adverse events. Stent occlusion did not occur in three of the four cases until the patients died of advanced cancer progression. EUS‐GJ using a 10 mm FCSEMS with a 7 Fr large‐loop double‐pigtail plastic stent or EUS‐HES is likely safe and effective for managing malignant afferent loop obstruction.

## INTRODUCTION

Recently, endoscopic ultrasound (EUS)‐guided gastrojejunostomy (EUS‐GJ) using a lumen‐apposing metal stent (LAMS) has been reported.^1^, ^2^, ^3^ LAMS can prevent stent migration and fluid leakage; however, it is unavailable in many countries and is more expensive than a conventional fully covered self‐expandable metal stent (FCSEMS).^4^

We performed EUS‐guided hepaticoenterostomy (EUS‐HES) and EUS‐GJ with a conventional FCSEMS, in patients with malignant afferent loop obstruction (m‐ALO) after Roux‐en‐Y reconstruction (RY), instead of EUS‐GJ with a LAMS. We present our experience with EUS‐guided treatments and evaluate the safety and efficacy of these procedures.

## CASE REPORT

Between February 2015 and February 2019, we treated four patients with m‐ALO after RY using EUS‐guided treatments. All procedures were performed using a convex‐type echoendoscope (GF‐UCT260; Olympus Medical Systems, Tokyo, Japan), a 19‐gauge needle, and a 0.025‐inch guidewire (VisiGlide 2; Olympus Medical Systems). All participants provided written informed consent, and the Kyoto Second Red Cross Hospital Institutional Review Board granted permission to review the patients’ records. The study was conducted according to the principles of the Declaration of Helsinki. Patient characteristics are summarized in Table 1. The symptoms improved without any adverse events in all cases.

**TABLE 1:** Patient characteristics

Case	Age/sex	Cause of ALO	Surgical procedure	EUS therapy	Procedure time (min)	AE	Time to stent dysfunction (days)	FU period after the procedure (days)
								
1	71/M	Recurrence of biliary cancer	Extrahepatic bile duct resection with CJ and RY	HGS	21	‐	32	212
2	69/M	Peritoneal dissemination because of ureteral cancer	Total gastrectomy with RY	HJS	20	‐	‐	51
3	77/F	Recurrence of pancreatic cancer	SSPPD with PG and RY	HGS	110	‐	‐	300
4	80/M	Recurrence of biliary cancer	SSPPD with PG and RY	GJ	57	‐	‐	17

Abbreviations: AE, adverse event; ALO, afferent loop obstruction; CJ, choledochojejunostomy; EUS, endoscopic ultrasound; FU, follow‐up; HGS, hepaticogastrostomy, HJS, hepaticojejunostomy; PG, pancreaticogastrostomy; RY, Roux‐en‐Y reconstruction; SSPPD, subtotal stomach‐preserving pancreaticoduodenectomy.

### Case 1

**FIGURE 1:**
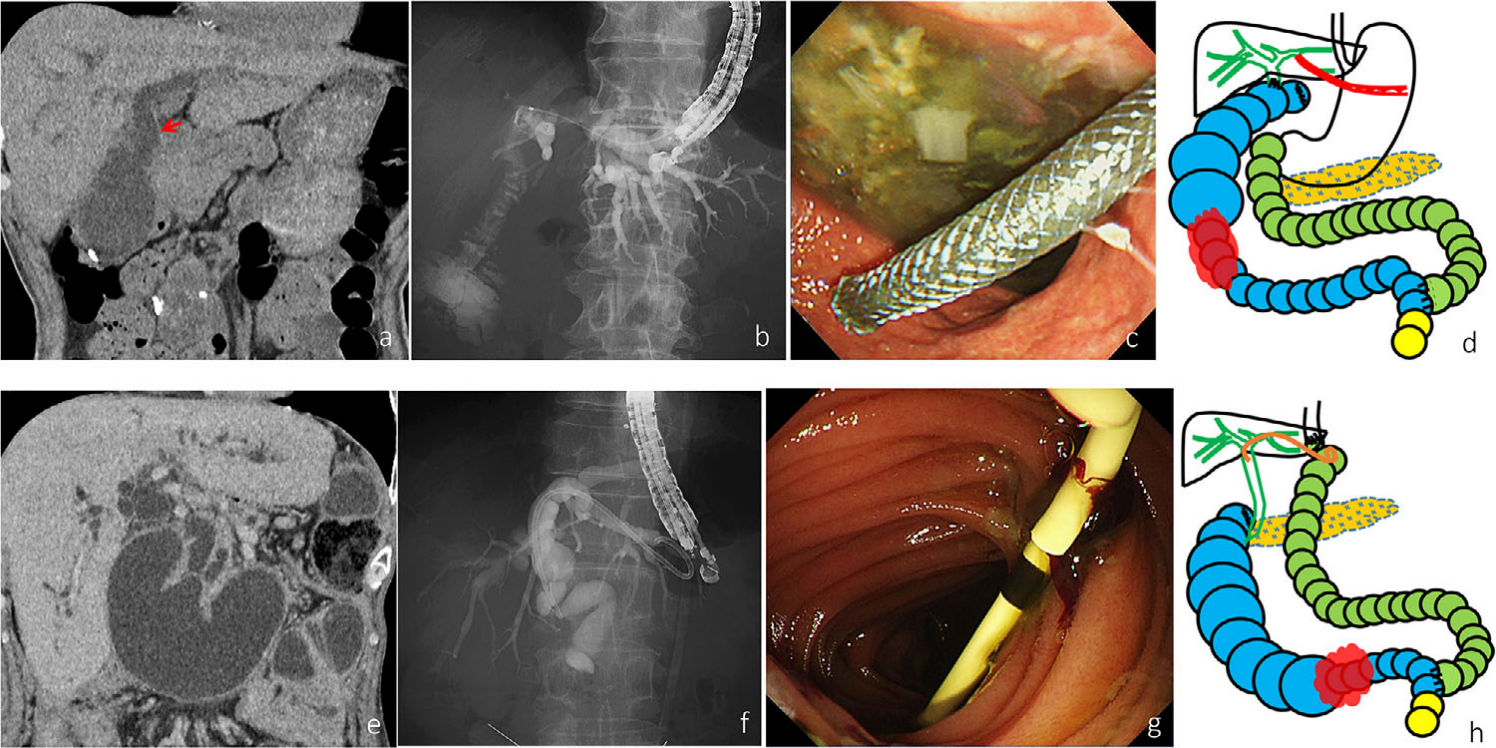
Case 1 (upper figure) and case 2 (lower figure). (a) Computed tomography showing the dilated afferent loop and intrahepatic bile duct. The choledochojejunostomy anastomosis was also dilated (red arrow). (b) Contrast medium was drained from the intrahepatic bile duct to the afferent loop through the choledochojejunostomy during the endoscopic ultrasound‐guided hepaticogastrostomy. (c) After the endoscopic ultrasound‐guided hepaticogastrostomy, the metal stent was seen in the stomach on endoscopic imaging. (d) A post‐stenting illustration. In the present case, bile juice and intestinal fluid will be drained out through the intrahepatic bile duct to the stomach. (e) Computed tomography showing a distended afferent loop and a dilated intrahepatic bile duct and main pancreatic duct. (f) Fluoroscopy during the endoscopic ultrasound‐guided hepaticojejunostomy showed that the plastic stent was placed from the common bile duct to the jejunum. (g) After the endoscopic ultrasound‐guided hepaticojejunostomy, the plastic stent was seen in the jejunum on endoscopic imaging. (h) A post‐stenting illustration. In the present case, bile juice, intestinal fluid, and pancreatic juice will be drained through the intrahepatic bile duct to the jejunum.

A 71‐year‐old male underwent extrahepatic bile duct resection with a choledochojejunostomy for distal biliary cancer. He was referred to us for cholangitis and obstructive jaundice caused by m‐ALO. Computed tomography (CT) revealed dilated afferent loop and intrahepatic bile duct (Figure 1a). Although a single‐balloon enteroscope (SBE) could reach the afferent loop stenosis (ALS), a guidewire could not be passed through. EUS‐GJ was not indicated because the dilated afferent loop was far from the stomach. We attempted EUS‐guided hepaticogastrostomy (EUS‐HGS) for bile and intestinal fluid drainage and successfully placed an 8 mm × 12 cm covered stent (bare‐end type, Niti‐S biliary S‐type; TaeWoong Medical, Seoul, Korea) from the left intrahepatic bile duct (segment 3) to the stomach (Figure 1b and c). He was discharged on post‐EUS‐HGS day 7 but readmitted on day 32 for acute cholangitis because of stent occlusion with biliary sludge. The sludge was aspirated using an endoscope, thus restoring bile flow. The patient died of cancer progression on post‐EUS‐HGS day 212.

### Case 2

A 69‐year‐old male who underwent total gastrectomy for gastric cancer 14 years ago was referred for obstructive jaundice caused by m‐ALO with peritoneal dissemination of ureteral cancer. CT showed a distended afferent loop and dilated bile and main pancreatic ducts (Figure 1e). An SBE could not reach the ALS. EUS‐guided jejunojejunostomy (EUS‐JJ) was not indicated because the dilated afferent loop was far from the jejunum. We attempted EUS‐guided hepaticojejunostomy (EUS‐HJS) and successfully placed a dedicated 8‐Fr plastic stent (Type IT; Gadelius Medical K. K., Tokyo, Japan) from the left intrahepatic bile duct (segment 3) to the jejunum (Figure 1f and g). CT on post‐EUS‐HJS day 6 showed improvement of the biliary duct, pancreatic duct, and afferent loop dilatation. He was discharged on post‐EUS‐HJS day 11. Stent occlusion did not occur until the patient died of cancer progression on post‐EUS‐HJS day 51.

### Case 3

**FIGURE 2:**
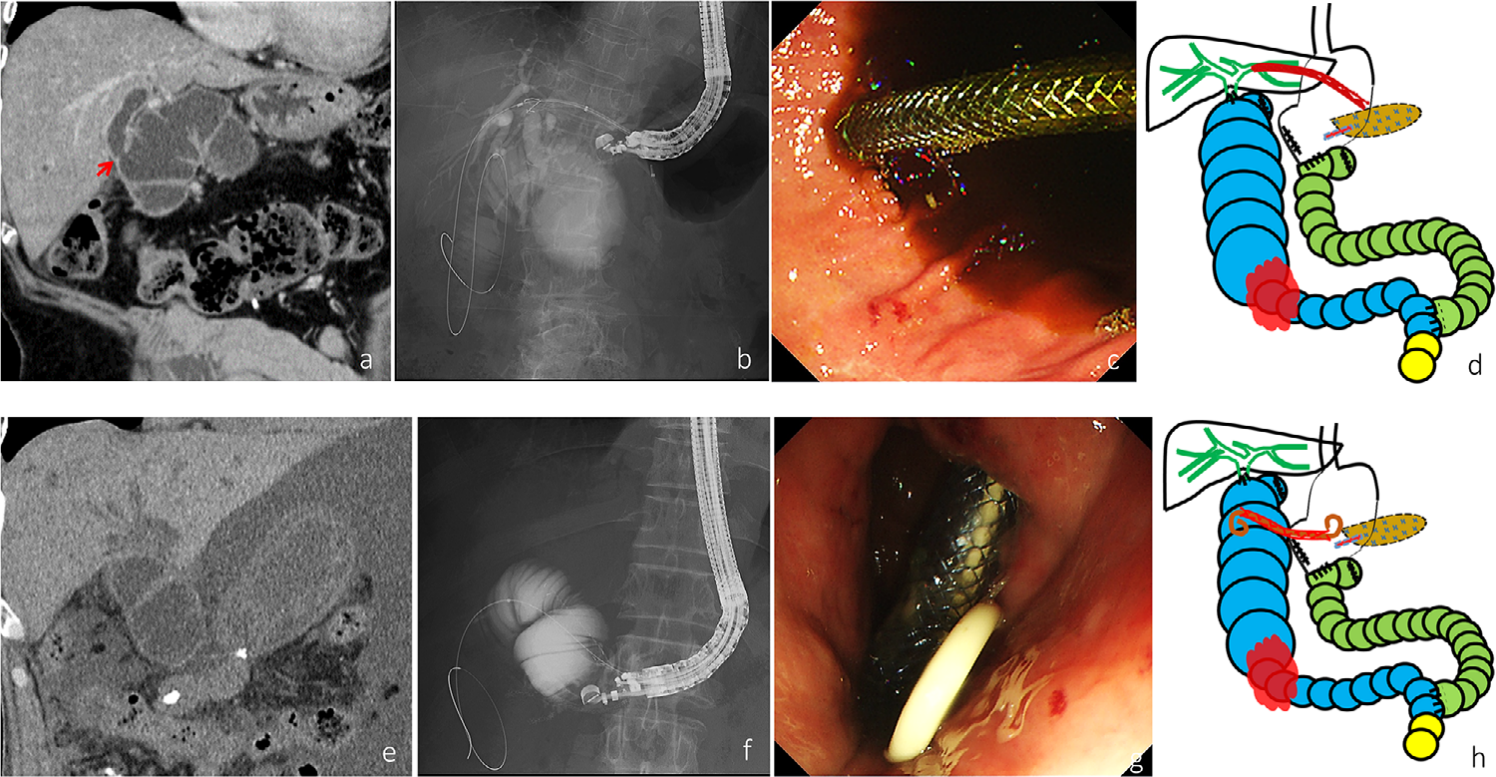
Case 3 (upper figure) and case 4 (lower figure). (a) Computed tomography showing the dilated afferent loop and intrahepatic bile duct. The choledochojejunostomy anastomosis was also dilated (red arrow). (b) Contrast medium was drained from the intrahepatic bile duct to the afferent loop through the choledochojejunostomy during the endoscopic ultrasound‐guided hepaticogastrostomy. (c) After the endoscopic ultrasound‐guided hepaticogastrostomy, the metal stent was seen in the stomach on endoscopic imaging. (d) A post‐stenting illustration. Bile juice and intestinal fluid will be drained out through the intrahepatic bile duct to the stomach, because a pancreaticogastrostomy was performed during the subtotal stomach‐preserving pancreaticoduodenectomy. (e) Computed tomography showing massive ascites between the liver and the stomach. (f) Fluoroscopy during the endoscopic ultrasound‐guided gastrojejunostomy showed the metal stent while deploying in the dilated afferent loop. (g) After the endoscopic ultrasound‐guided gastrojejunostomy, the metal stent with the plastic stent was seen in the stomach on endoscopic imaging. (h) A post‐stenting illustration. Bile juice and intestinal fluid will be drained out through the stent to the stomach because a pancreaticogastrostomy was performed during the subtotal stomach‐preserving pancreaticoduodenectomy.

A 77‐year‐old female who underwent a subtotal stomach‐preserving pancreaticoduodenectomy for pancreatic cancer was referred for obstructive jaundice caused by m‐ALO. CT showed a dilated afferent loop and intrahepatic bile duct (Figure 2a). An SBE could not reach the ALS, and EUS‐guided treatment was attempted. Although EUS showed dilated afferent loop and intrahepatic bile duct from the stomach, we attempted EUS‐HGS because a LAMS was not yet available in Japan for EUS‐GJ. Although insertion of guidewire toward the hepatic hilum took longer, we successfully placed an 8 mm × 12 cm covered stent (Niti‐S biliary S‐type) from the left intrahepatic bile duct (segment 2) to the stomach (Figure 2b and c). She was discharged on post‐EUS‐HGS day 8. Stent occlusion did not occur until she died of cancer progression on post‐EUS‐HGS day 300.

### Case 4

An 80‐year‐old male who underwent a subtotal stomach‐preserving pancreaticoduodenectomy for distal biliary cancer was referred for cholangitis and obstructive jaundice caused by m‐ALO. He underwent surgery for adhesive intestinal obstruction near the jejunojejunostomy anastomosis of the afferent loop 4 months ago. Because of massive ascites caused by peritoneal dissemination and the high possibility of strong adhesion near the afferent loop, insertion of the SBE into deep small intestine was considered challenging and the risk of intestinal perforation was high. EUS‐HGS was not indicated because of massive ascites between the liver and the stomach (Figure 2e). There were no intervening ascites between the stomach and the dilated afferent loop on EUS, and EUS‐GJ was attempted. After the dilated afferent loop was punctured from the stomach, a guidewire was inserted and coiled into the afferent loop. The needle tract was dilated using a 4 mm balloon catheter (REN; Kaneka Medix, Osaka, Japan), and a 10 mm × 8 cm FCSEMS (BONA stent; Standard Sci‐Tech Inc., Seoul, Korea) was deployed from the afferent loop to the stomach. A 7 Fr × 10 cm large‐loop double‐pigtail plastic stent (Double Pigtail; Medi‐Grobe GmbH, Germany) was placed through the FCSEMS to prevent stent migration and dislocation (Figure 2f, g, and h). CT on post‐EUS‐GJ day 8 revealed appropriate placement of the stent. The patient died of cancer progression on post‐EUS‐GJ day 17.

## DISCUSSION

M‐ALO after RY is conventionally treated with percutaneous drainage, surgery, or endoscopic enteral stenting.^5^ Although previous reports described percutaneous transhepatic enteral stent insertion and direct percutaneous tube enterostomies as useful,^6^, ^7^ percutaneous treatment may affect patients’ quality of life during external drainage tube placement. The difficulty of reoperation and advanced cancer stage make surgery challenging.^5^ Although previous reports described the usefulness of endoscopic stent placement across malignant afferent stenosis,^8^, ^9^ scope insertion is often difficult because of postoperative adhesion, peritoneal dissemination, and/or ascites.

We have reported four cases of m‐ALO after RY: three patients underwent EUS‐HES (two EUS‐HGS and one EUS‐HJS) and one patient underwent EUS‐GJ with conventional biliary FCSEMS. In cases 1 and 2, neither EUS‐GJ nor EUS‐JJ was indicated because the dilated afferent loop was far from the stomach or the jejunum; instead, EUS‐HES was performed. EUS‐GJ was an option in case 3, but EUS‐HES was performed because a LAMS was unavailable for EUS‐GJ in Japan. Further investigations are required to determine the feasibility of EUS‐HES or EUS‐GJ (or EUS‐JJ) when both dilated afferent loops and intrahepatic bile ducts are visualized by EUS, as in case 3. In case 4, EUS‐GJ was performed because EUS‐HES was not indicated due to massive ascites around the liver. A 10 mm FCSEMS combined with a 7 Fr large‐loop double‐pigtail plastic stent was placed to prevent stent migration and dislocation. This stent system may be an option for EUS‐GJ for m‐ALO.^4^, ^10^

In cases 1 and 3, after EUS‐HES, bile juice and intestinal fluid were drained through the intrahepatic bile duct into the stomach (Figures 1d and 2d). However, in case 2, after EUS‐HES, pancreatic juice accumulated in the afferent loop was also drained through the intrahepatic bile duct into the jejunum (Figure 1h). Although this situation can create problems such as the development of biliary cancer in the long term, we believe that these procedures are permissible for treating m‐ALO.

In case 2, although the dilatation of the pancreatic duct and afferent loop had improved, balloon dilatation for the papilla or stent placement across the papilla, combined with EUS‐HES, could have been better for smooth drainage of the intestinal and pancreatic fluid accumulated in the afferent loop.

In all four cases, the patients’ symptoms improved without adverse events. Stent occlusion did not occur in three of the cases until the patients died of cancer progression.

In conclusion, our experience suggests that EUS‐GJ using a 10 mm FCSEMS with a 7 Fr large‐loop double‐pigtail plastic stent or EUS‐HES is safe and effective for managing m‐ALO; however, further large‐scale investigations are needed to confirm our findings.

## CONFLICT OF INTEREST

Authors declare no conflict of interest for this article.

## FUNDING INFORMATION

None.
